# Uncovering miRNAs involved in crosstalk between nutrient deficiencies in *Arabidopsis*

**DOI:** 10.1038/srep11813

**Published:** 2015-07-02

**Authors:** Gang Liang, Qin Ai, Diqiu Yu

**Affiliations:** 1 Key Laboratory of Tropical Forest Ecology, Xishuangbanna Tropical Botanical Garden, Chinese Academy of Sciences, Kunming, Yunnan 650223, China; 2 University of Chinese Academy of Sciences, Beijing 100049, China

## Abstract

Integrating carbon (C), nitrogen (N), and sulfur (S) metabolism is essential for the growth and development of living organisms. MicroRNAs (miRNAs) play key roles in regulating nutrient metabolism in plants. However, how plant miRNAs mediate crosstalk between different nutrient metabolic pathways is unclear. In this study, deep sequencing of *Arabidopsis thaliana* small RNAs was used to reveal miRNAs that were differentially expressed in response to C, N, or S deficiency. Comparative analysis revealed that the targets of the differentially expressed miRNAs are involved in different cellular responses and metabolic processes, including transcriptional regulation, auxin signal transduction, nutrient homeostasis, and regulation of development. C, N, and S deficiency specifically induced miR169b/c, miR826 and miR395, respectively. In contrast, miR167, miR172, miR397, miR398, miR399, miR408, miR775, miR827, miR841, miR857, and miR2111 are commonly suppressed by C, N, and S deficiency. In particular, the miRNAs that are induced specifically by a certain nutrient deficiency are often suppressed by other nutrient deficiencies. Further investigation indicated that the modulation of nutrient-responsive miRNA abundance affects the adaptation of plants to nutrient starvation conditions. This study revealed that miRNAs function as important regulatory nodes of different nutrient metabolic pathways.

Plants often grow in soil that contains very low concentrations of macronutrients or suffer from an unbalanced supply of macronutrients. To adapt and grow in nutrient-deprived environments, plants have developed strategies to cope with different nutrient ion limitations. When plants encounter starvation of a certain mineral nutrient, the metabolism of other nutrients will be adjusted to maintain proper growth and development. Previous studies suggested that the balance between carbon (C) and nitrogen (N), rather than one single metabolite, affects global gene expression. In addition, cellular C and N metabolism is tightly coordinated for C-N-containing metabolite synthesis at the biochemical level and for long-distance sensing and signaling of the C/N balance^1^. Microarray-base transcript analysis suggested that both N starvation^2^ and N-resupply after starvation^3^,^4^ affect many genes involved in C or S metabolism. Direct testing of the interactive effects of C/N on genome-wide gene expression supported the view that more than half of *Arabidopsis*’ genes are either induced or suppressed by C alone, N alone, or C-N combinations^5^,^6^,^7^.

After entering the cell, sulfate is activated and reduced, and then incorporated into cysteine. The formation of cysteine involves the conjugation of sulfide with *O*-acetylserine (OAS), which is produced through the acetylation of serine. Provision of serine depends on adequate C and N metabolism, and this convergence node of the assimilation pathways represents an opportunity for the coordination of S assimilation with C and N metabolism. When plants were fed with sucrose or glucose, the key enzyme of sulfate reduction, APS reductase was stimulated^8^,^9^,^10^. The sulfate transporters, SULTR1;1 and SULTR1;2, are mainly responsible for sulfate uptake from soil to the roots^11^. The mRNA levels of *SULTR1;1* and *SULTR1;2* were substantially increased in plants grown with sucrose under S-deficient (–S) conditions^12^. In contrast, removing sucrose from the media considerably attenuated the induction of *SULTR1;1* and *SULTR1;2* by –S^13^. These results suggested that the assimilation and transport of sulfate are not only regulated by the S status, but also controlled by the fluctuation of the C status. In addition to the influences of the C status, the expression of *SULTR1;1* and *SULTR1;2* was also significantly regulated by the N status. Similar to the contribution of C starvation, the reduction of the N supply significantly suppressed the induction of *SULTR1;1* and *SULTR1;2* by S depletion^13^. In contrast, the sulfate deprivation disrupted N metabolism, resulting in high levels of nitrate in leaves^14^.

Amino acids are necessary for the synthesis of proteins, which maintains the whole life cycle of plants. N and C are essential for the synthesis of amino acids and S-containing Met is required for protein synthesis. Therefore, the coordination of C, N, and S metabolism ensures the fitness and propagation of plants. In fact, in addition to the interaction between the C, N, and S metabolisms, other nutrient metabolisms also interact with C, N, and S assimilation, such as phosphorus (P) and potassium (K)^15^,^16^,^17^,^18^,^19^. Despite genome-wide gene expression analyses, the mechanism underlying the crosstalk among C, N, and S metabolic pathways is unclear, as is the crosstalk of C/N/S with other nutrient metabolic pathways.

Plant microRNAs (miRNAs) are a particular class of small RNAs that mainly participate in gene regulation at the post-transcription level. miRNAs are processed from endogenous mRNA with a stem-loop structure by Dicer-like proteins, which are further incorporated into RISC (RNA induced Silencing Complex) and then recruited to their target mRNAs by complementary base-pair. At last, miRNAs mediate the cleavage or (and) translation repression of target mRNAs^20^. Several miRNAs involved in nutrient metabolic pathways have been characterized. For example, miR395 and miR399 mediate the regulation of sulfate and phosphate homeostasis, respectively^21^,^22^. Copper (Cu) starvation induces miR397, miR398, miR408, and miR857 and mediates the downregulation of copper proteins^23^,^24^. miR169 and miR826 oppositely regulate N starvation response^25^,^26^. miR827 plays pivotal roles in regulating phosphate homeostasis in plants in a nitrate-dependent fashion^27^. In addition, deep sequencing identified some nutrient-responsive miRNAs, such as miR778, miR828, and miR2111, which were induced during phosphate limitation^28^,^29^.

To cope with nutrient-deprived environments, plants must sense changes in the external and internal mineral nutrient concentrations and adjust their nutrient metabolisms to meet the demands of plant growth. miRNAs are enriched in the vascular tissues^30^,^31^ where the translocation and distribution of nutrients occurs. miR399 is a long-distance signal for phosphate homeostasis^32^. Therefore, nutrient-responsive miRNAs might play crucial roles in modulating nutrient starvation responses and crosstalk among nutrient metabolic pathways. To date, how plant miRNAs mediate the interaction and crosstalk among different nutrient metabolisms remains unknown. This study systemically analyzed the expression of miRNAs under C, N, or S starvation conditions. Some miRNAs are specifically responsive to a specific nutrient deficiency, and others are regulated differentially by different nutrient deficiency conditions. This study confirmed that a large number of miRNAs are responsive to nutrient deficiencies and some of them mediate the interaction between different nutrient metabolic pathways.

## Results

### Overview of small RNA profiles

**Figure 1:**
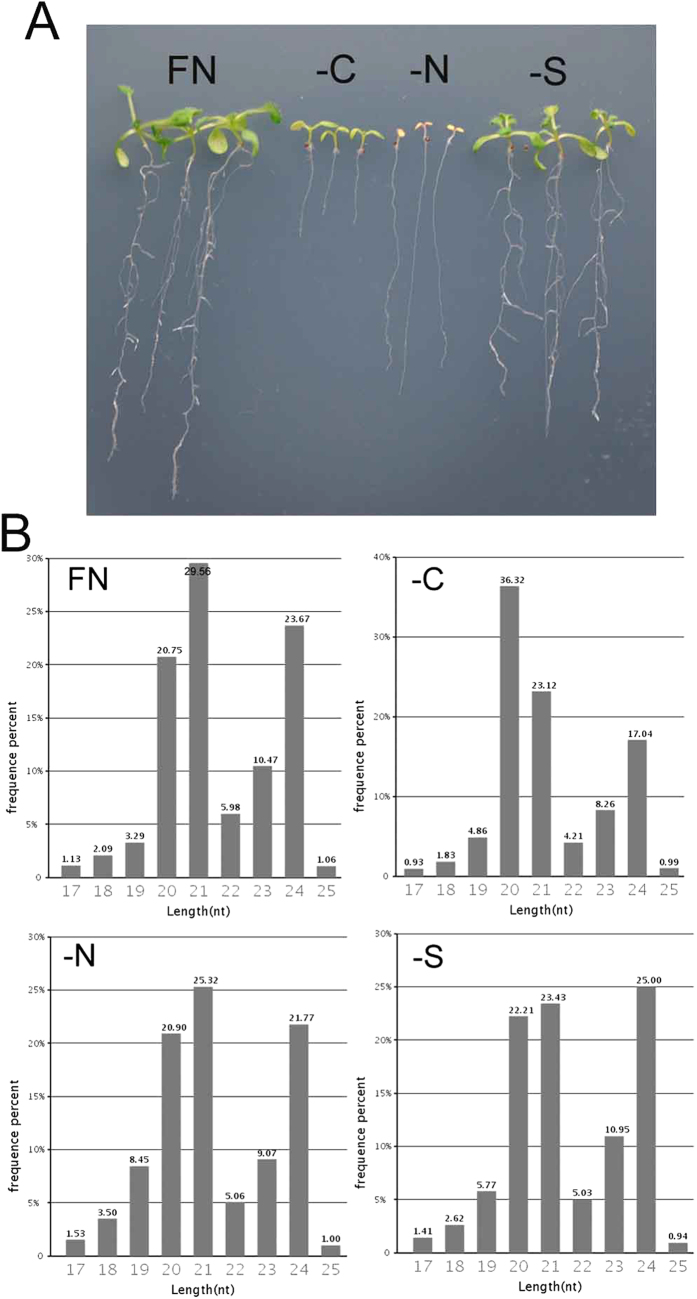
Responses of seedlings and small RNAs to nutrient deficiency. (**A**). Ten-day-old seedlings grown under full nutrient (FN) and nutrient deficiency (–C, –N, and –S) conditions. (**B**). Abundance of small RNA sequences with different sizes.

To characterize the small RNAs that are responsive to C, N, or S deficiency, 10-day-old seedlings grown on full nutrient (FN), sucrose-free (–C), nitrogen-free (–N), or sulfate-free (–S) MS medium were used to construct small RNA libraries. Compared with the FN seedlings, nutrient-deficient seedlings displayed significant nutrient starvation symptoms. –C seedlings produced short primary roots; –N seedlings displayed significantly delayed leaf growth; and –S seedlings gave produced long lateral roots (Fig. 1A).

By deep sequencing on the Solexa platform, 9,681,350; 10,860,761; 10,285,363; and 9,810,707 reads were generated from FN, –C, –N, and –S libraries, respectively. After removing adaptor sequences and low-quality reads, the numbers of sequence reads and unique sequence from the raw data were calculated and then mapped to the *Arabidopsis thaliana* genome. More than 85% of total sequences were perfectly mapped to the genome, which were further clustered into 11 classes, including exon_antisense, exon_sense, intron_sense, intron_antisense, miRNA, rRNA, repeat, snRNA, snoRNA, tRNA and unannotation (Table 1).

The mapped sequences with lengths ranging from 17 to 25 nucleotides were used to examine the correlation between the length of small RNAs and the proportion of total sequence reads. The most abundant size of small RNAs in the FN library was 21 nucleotides (29.56%), followed by 24 nucleotides (23.67%). The small RNA size distribution pattern in –N was similar to those in FN. In contrast, the small RNAs in –C and –S displayed a different distribution pattern; 20 and 24-nucleotide-length small RNAs were the most abundant in –C and –S, respectively (Fig. 1B). These results suggested that different nutrient supplies caused differential distribution patterns of the sizes of small RNAs.

### Profile of known miRNAs

To calculate the number of known miRNAs in each library, we aligned all the unique sequences with lengths between 19 and 26 to all annotated miRNA precursor sequences in *Arabidopsis* (miRbase Release 18.0, www.miRbase.org). In total, 3,909,038; 5,344,206; 3,968,586; and 3,378,379 mapped sequences were identified for each library, respectively. To compare the miRNA abundance in the different libraries, the abundance of each miRNA in a library was normalized to transcripts per million (TPM) to represent the relative read frequency in each library. miRNAs with an abundance <5 TPM in each library were excluded. The remaining 133 unique mature miRNAs belonged to 60 miRNA families (Supplemental Table 1). In all four libraries, the miR156 family was the most abundant, followed by the miR167 and miR166 families. Out of the 60 miRNA families, 28 families have more than two distinct miRNA sequences. Unlike northern blotting, deep sequencing can be used to analyze different miRNA species from the same family. Based on the sequencing results, the major differences in abundance for different miRNA members from the same family were calculated, such as for the miR156, miR158 and miR159 families. Differential responses of miRNA members to the same nutrient deficiency were also revealed. For example, miR164a and miR164b were downregulated 14-fold by –C, whereas miR164c was not responsive to –C. Similar cases also occurred in the miR169 family and others. These observations implied that different members from the same family might play distinct functions in response to nutrient deficiencies.

### miRNAs responsive to a specific nutrient deficiency

**Figure 2:**
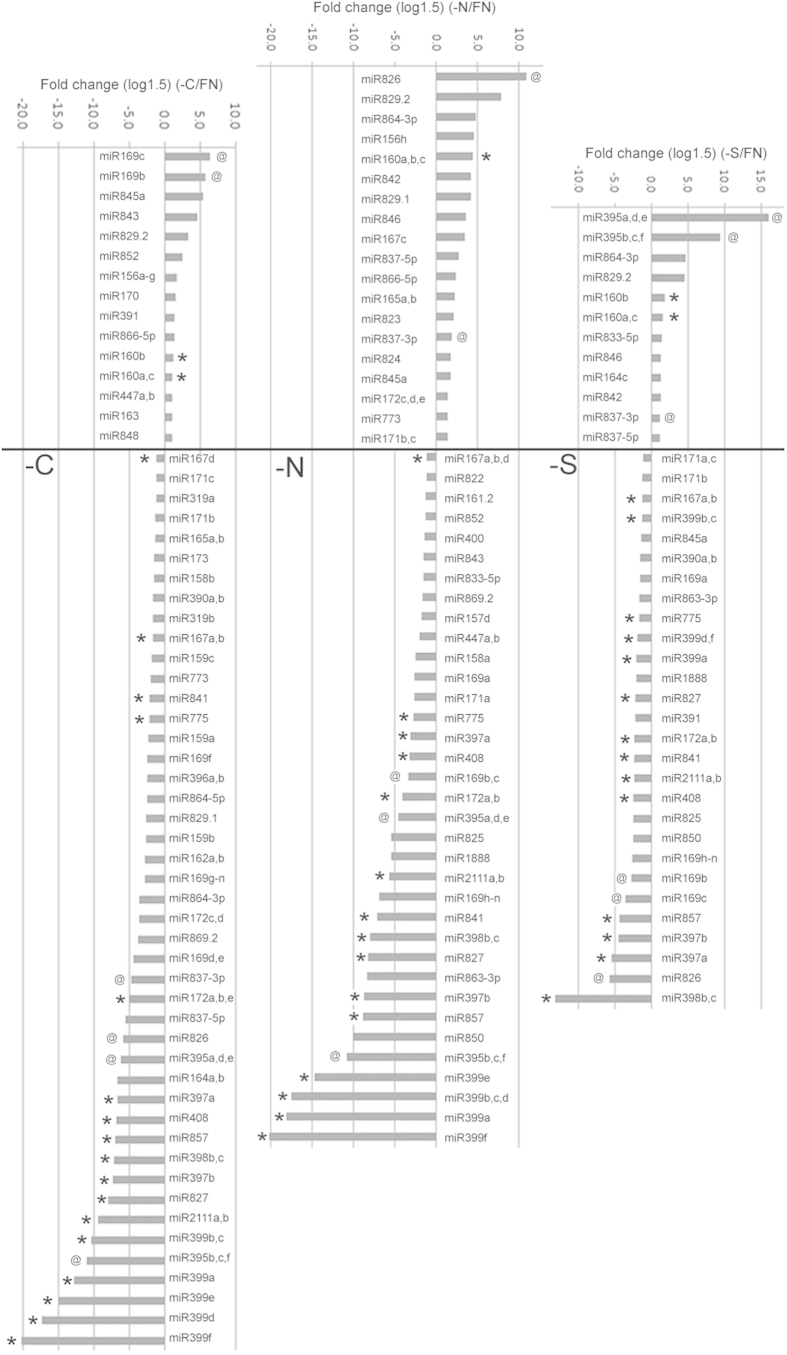
Differentially expressed miRNAs in response to –C, –N, and –S. The significantly differentially expressed miRNAs (greater than 1.5-fold relative change) are shown. The bars marked by one ‘*’ indicate that the miRNAs are repressed by all three types of nutrient deficiencies (–C, –N, and –S). The bars marked by one ‘@’ indicate that the miRNAs are induced by one of the three types of nutrient deficiencies, but repressed by the other two. The bar shared by two or more miRNA members indicates that these miRNAs have the same read number.

To explore the miRNAs that were differentially expressed in response to a specific nutrient deficiency, we compared the read counts of miRNAs under nutrient-deficient conditions with those under FN. The miRNAs with greater than 1.5-fold relative change in sequence counts were identified as differentially expressed miRNAs (Fig. 2). Ninety-two, 79, and 59 differentially expressed miRNAs, which are clustered into 40, 41 and 31 miRNA families, were obtained for –C, –N, and –S, respectively (Supplemental Table 1).

miR163, miR169b/c, miR170, miR391, miR447, miR843 and miR848 were specifically upregulated, whereas miR159, miR162, miR164a/b, miR165, miR169d–g, miR172c/d, miR173, miR319, miR773, and miR864-3p were specifically downregulated by –C conditions. Under –N conditions, miR165, miR167c, miR171b/c, miR172c–e, miR773, miR823, miR824, miR826, miR829.1, and miR842 were induced specifically, whereas miR157d, miR158a, miR161.2, miR400, miR447, miR822, miR833-5p, miR843, and miR852 were suppressed. In contrast, fewer differentially expressed miRNAs were identified under –S conditions: miR164c and miR395 were upregulated specifically, and miR391 and miR845a were downregulated. Although there have been no reports about –C-responsive miRNAs, previous studies on –N and –S-responsive miRNAs showed that miR169 and miR395 are negatively and positively responsive to –N and –S, respectively^21^,^25^. Given that miR169 members displayed differential responses to –N, we calculated all miR169 mature sequence counts, which revealed that the total number of miR169 mature sequences in –N was two-fold less than that in FN (Supplemental Table 2). miR395 was induced sharply by –S. Both downregulation of miR169 by –N and upregulation of miR395 by –S were consistent with previous reports, confirming the reliability of the sequencing data.

To investigate the putative functions of these specifically nutrient-responsive miRNAs, we analyzed their target genes (Supplemental Table 4). Most of the targets are involved in development, stress responses and nutrient metabolism, which are closely linked with nutrient supply conditions.

### miRNAs commonly responsive to –C, –N, and –S

To identify the miRNAs that are commonly responsive to all three nutrient deficiencies, we compared differentially expressed miRNAs among –C, –N, and –S: 16 miRNA families were identified (Fig. 2 and Table 2). The responses of these miRNAs to various nutrient deficiencies were further confirmed by real-time PCR (Supplemental Table 3). miRNA members from 11 miRNA families were downregulated, whereas one family was upregulated, by –C, –N, and –S. Among the 11 downregulated miRNA families, eight are directly associated with metabolic process. For example, miR398, miR397, miR408 and miR857 target *Superoxide Dismutase* (*CSD1*, *CSD2*, and *CCS1*), *Cytochrome oxidase c, Laccase* (*LAC2/3/4/7/12/13/17*), and *Plantacyanin* to regulate the copper starvation (–Cu) response and homeostasis^24^,^33^. miR399, miR827, and miR2111 play key roles in the phosphate starvation response and homeostasis. miR399 targets *PHO2* which encodes a ubiquitin E2 conjugase and regulates the allocation of phosphate^22^,^34^. The target of miR827 is *NLA* (*Nitrogen Limitation Adaptation*), the product of which mediates degradation of plasma membrane-localized phosphate transporters to maintain phosphate homeostasis in *Arabidopsis*^35^. miR2111 is specifically induce by P limitation and its target gene is a *Kelch repeat-containing F-box* gene with unknown function^28^. miR775 functions in secondary metabolite biosynthesis by targeting a gene that encodes a galactosyltransferase^36^. In contrast, among the remaining three repressed miRNA families, miR167 and miR172 participate in the auxin response^37^ and the juvenile-to-adult transition^38^, respectively, and the function of miR841 is unknown. The only miRNA family induced commonly by –C, –N, and –S was miR160, which is involved in the auxin response by targeting *ARF10*, *ARF16* and *ARF17*^39^,^40^.

In contrast to the miRNAs that were regulated similarly by –C, –N, and –S, miR169b/c, miR395, miR822, and miR837-3p were regulated differentially by –C, –N, and –S. miR169b/c was induced by –C, but suppressed by –N and –S. miR395 was positively regulated by –S, but negatively by –C and –N. miR826 was upregulated by –N, but downregulated by –C and –S. miR837-3p decreased in response to –C, but increased to –N and –S. Interestingly, the three miRNA families (miR399, miR827, and miR2111) induced by phosphate starvation (–P) were also repressed by other nutrient deficiencies (–C, –N, and –S). This result implied that miRNAs induced specifically by one nutrient deficiency are suppressed by other nutrient deficiencies. To confirm whether this case also occurs for other miRNAs that are induced by other specific nutrient deficiency, we retrieved and analyzed the small RNA sequencing data generated under –P conditions^28^. Although the miR826 sequence was not found because of low expression abundance in –P, we found that –C-induced miR169b/c, –S-induced miR395, and –Cu-induced miRNAs (miR397, miR398, miR408, and miR857) were significantly suppressed in roots grown in –P conditions (Supplemental Table 5). The interaction between different nutrient metabolisms is common and involves a large number of genes in plants. Our results indicated that many miRNA families that directly participate in the regulation of certain specific nutrient metabolism processes were differentially expressed in response to different nutrient deficiencies. Therefore, it is likely that nutrient-responsive miRNAs also mediate the crosstalk between different nutrient metabolism processes.

### Expression correlation between miRNAs and their targets

**Figure 3:**
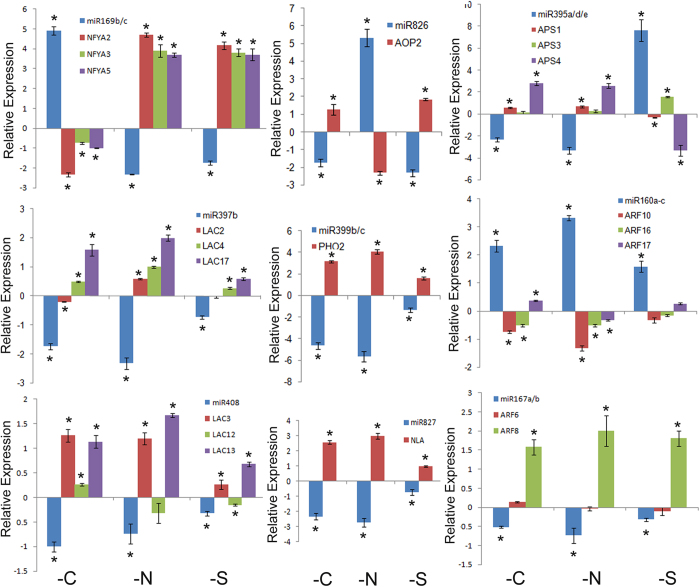
Expression of miRNAs and their targets in response to nutrient deficiencies. RNA was isolated from 10-day-old seedlings. Relative expression was indicated by the log2 value. Student’s t test indicated that the values marked by one asterisk are significantly different from the corresponding full nutrient value (P < 0.01; n = 3).

Plant miRNAs mediate target mRNA cleavage or translation inhibition; therefore, the expression of plant miRNAs is usually negatively correlated with that of their targets. To test this, the expression patterns of miRNAs and their targets were compared using quantitative RT-PCR assays. As expected, most targets displayed the opposite expression trends compared with the corresponding miRNAs. For example, miR169b/c, miR826, and miR395a/d/e were induced by –C, –N, and –S, respectively, and their targets were repressed correspondingly, with the exception of *APS3* (Fig. 3A–C). The expression patterns of miR826-*AOP2* in –N and miR395-*APS1/3/4* in –S agreed with the previous reports^21^,^26^,^41^. miR397 and miR408 are involved in the copper starvation response. Consistent with the sequencing data, their expression was downregulated by –C, –N, and –S. Among the six target genes examined, the expressions of *LAC4* and *LAC17* were inversely related to miR397b (Fig. 3D), as were those of *LAC3* and *LAC13* to miR408 (Fig. 3G). Both miR399 and miR827 were suppressed by –C, –N, and –S, and their targets, *PHO2* and *NLA*, were induced correspondingly (Fig. 3E,H). miR160 and miR167 are involved in the auxin pathway by targeting *ARF* genes. miR160 was induced whereas miR167a/b was repressed by –C, –N, and –S. In contrast, the targets of miR160, *ARF10* and *ARF16*, were repressed, whereas the target of miR167, *ARF8*, was induced by –C, –N, and –S (Fig. 3F,I). In addition, some miRNAs were positively correlated with their targets, such as miR395-*APS3* in –S, miR397-*LAC2* in –C, and miR160-*ARF17* in –C and –S. It is likely that these targets are also regulated by other transcription factors or their expression does not completely overlap with miRNAs spatiotemporally.

### Altered miRNA expression caused differential adaptation to nutrient deficiency

**Figure 4:**
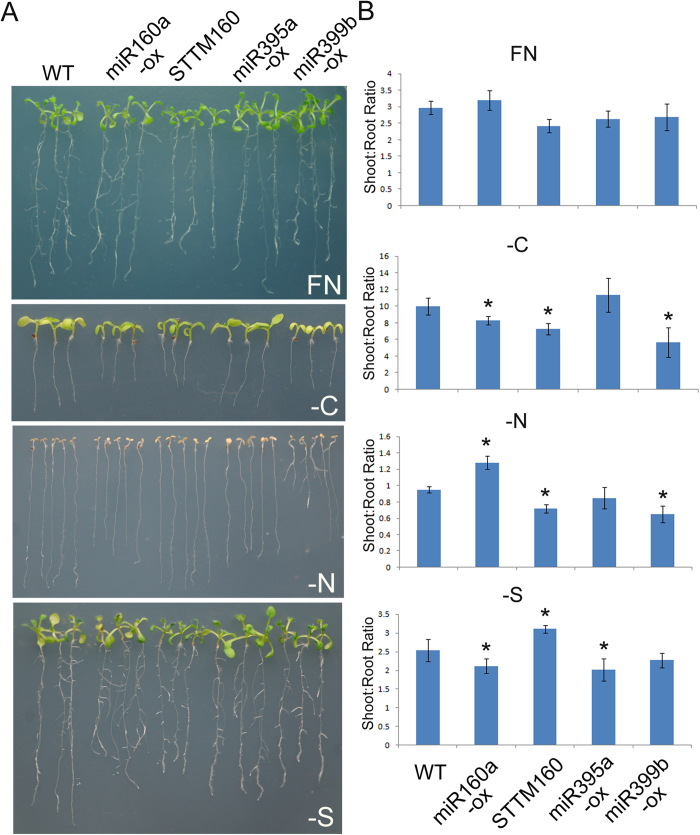
Phenotypes of transgenic plants under nutrient starvation conditions. (**A**) Ten-day-old seedlings grown vertically. (**B**) The ratio of shoot/root mass of 10-day-old seedlings grown under full nutrient and nutrient-deficient conditions. The error bars represent the SD from triplicate samples with each sample containing 10–15 plants. Student’s t test indicated that the values marked by one asterisk are significantly different from the corresponding full nutrient value (P < 0.01; n = 3).

Overexpression of a certain nutrient-responsive miRNA altered the plant’s adaptation to nutrient starvation conditions^21^,^22^,^25^,^27^. To investigate the functions of nutrient-responsive miRNAs in nutrient starvation adaptation, miRNA overexpression plants (miR160a-ox, miR395a-ox, and miR399b-ox) and miR160 suppression plants (STTM160) were used to evaluate phenotypes in nutrient starvation conditions. As shown in Fig. 4A, miR160a-ox, STTM160, and miR399b-ox plants produced short primary roots compared with wild-type plants under –C conditions. Under –N conditions, miR399b-ox plants developed lateral roots, whereas the wild-type and the other transgenic plants had no visible lateral roots. Under –S conditions, the lateral roots of miR160-ox and miR395a plants were longer than the other plants. The shoot:root ratio is one measure to assess the growth status of plants. The shoot:root ratio of miR160a-ox plants was lower under –C and –S conditions, but higher under –N conditions compared with the other plants. STTM160 and miR399b plants had lower shoot:root ratios than the other plants under –C and –S conditions. In contrast, miR395a-ox plants only displayed a differential shoot:root ratio under –S conditions. These results suggested that misexpression of nutrient-responsive miRNAs leads to altered adaptation to nutrient deficiency.

## Discussion

### Specificity of miRNA regulation under nutrient-deficient conditions

Although a large number of miRNAs were differentially expressed in response to nutrient deficiencies, some miRNAs were specifically responsive to specific nutrient depletions. miR169b/c, miR826, and miR395 showed the largest changes in response to –C, –N, and –S, respectively. Our previous studies confirmed that miR826 regulates the N starvation adaptation of Arabidopsis by reducing glucosinolate synthesis^26^, and miR395 mediates S homeostasis by regulating sulfate assimilation and transport^21^. Despite the implication of miR169 in N starvation response, miR169b/c was induced specifically by –C, implying that miR169b/c plays a particular role in C starvation response. Further discussion on miR169 is provided below. Recently, miR156, which mediates juvenile-to-adult phase transition, was confirmed to be suppressed by the addition of sucrose^42^. Correspondingly, we found that miR156 (except for miR156 h) was specifically induced by the depletion of sucrose. It implies that C status directly affects the abundance of miR156.

Notably, two negative regulators of leaf senescence, miR164a/b and miR319, were specifically repressed by –C. Leaf senescence allows for the degradation of the nutrients produced during the growth phase of the leaf and their reallocation to developing tissues or organs to maximize the fitness of the plant^43^. miR164, whose expression gradually decreased with leaf aging, targets *NAC2/ORE1*, which functions positively in leaf senescence^44^. The miR319-regulated clade of *TCP* transcription factor genes facilitates the biosynthesis of the hormone jasmonic acid, which then accelerates leaf senescence^45^. It is likely that carbon starvation induces leaf senescence by suppressing the expression of miR164 and miR319. Although leaf senescence is a common symptom induced by nutrient deficiencies, the expression of both miRNAs was not responsive to –N and –S. This suggested that leaf senescence regulation mediated by miR164 and miR319 is specific to –C, whereas –N and –S induce leaf senescence by other regulatory pathways.

C and N metabolites can function as signals to influence many cellular processes by regulating gene expression in plants. C and N metabolites participate in developmental processes, including flowering time^46^, root architecture modulation^47^ and several metabolic pathways (e.g., N assimilation and amino acid synthesis)^48^,^49^. A previous study confirmed that a complex C/N-responsive gene network exists in plants, and the balance between C and N affects global gene expression^7^. Under –C conditions, miR447 was induced, whereas –N conditions suppressed miR447. The direct target of miR447 encodes a p-loop containing nucleoside triphosphate hydrolase, which is involved in secondary metabolite biosynthesis^50^. This result implied that the C-N balance mediates regulation of secondary metabolite biosynthesis by affecting the expression of miR447. In addition, the responses of miR165, miR172c/d/e, and miR773 to C starvation were opposite to that to N starvation. Under N limitation conditions, miR165, miR172c/d/e and miR773 were upregulated whereas under C starvation conditions, they were downregulated. miR165 affects shoot apical meristem development via the regulation of HD-ZIP III genes^51^,^52^; miR172 mediates juvenile-to-adult transition by the suppression of several *AP2* transcription factors^53^; and miR773 targets a gene that encodes a DNA methyltransferase^36^. Therefore, it is likely that these three miRNAs mediate the growth and development of plants in response to the C and N starvation.

In addition to conserved miRNAs, some *Arabidopsis*-specific miRNAs were also involved in the response to nutrient deficiencies (Supplemental Table 4). For example, miR163, miR773, miR843, miR848, and miR854-3p were responsive to –C; miR158a, miR161.2, miR400, miR447, miR773, miR822, miR823, miR826, miR833-5p, miR843, and miR852 were responsive to –N; and miR845a was responsive to –S. Although the targets of most *Arabidopsis*-specific miRNAs are unknown, the target genes of miR158, miR163, and miR826 encode glycosyltransferase, methyltransferases, and 2-oxoglutarate-dependent dioxygenase, all of which play roles in secondary metabolite biosynthesis^26^,^41^,^54^. This result implied that nutrient deficiencies affect the biosynthesis of secondary metabolites indirectly by altering abundance of miRNAs.

### Universality of miRNA regulation under nutrient-deficient conditions

miR169 was repressed by –N and regulated nitrogen homeostasis in *Arabidopsis*^25^. Interestingly, the total number of miR169 mature sequences was also downregulated by –S (Supplemental Table 2). A similar repression of miR169 was observed under –P conditions in *Arabidopsis*^28^. These observations suggested that miR169 might not only have a specific function in nitrogen homeostasis, but also in S and P metabolism. Notably, different miR169 members showed differential responses to –C, –N, and –S, as well as to –P (Supplemental Table 2 and 5). With other miR169 species unaffected or reduced, miR169b/c was dramatically induced by –C. A previous study revealed that –N specifically upregulated miR169d–g, but not other miR169 species^21^. In contrast, –S and –P suppressed nearly all miR169 species. Therefore, it is likely that miR169b/c and miR169d–g specifically affect C and N metabolism, respectively. There are one or two nucleotide differences between miR169 species, implying that different miR169 mature sequences may have different target genes, as described in a recent study, which confirmed that miR157d, but not other species of the miR156/157 family, mediated the cleavage of the *HY5* mRNA^55^.

Nutrient availability is closely linked with root development. Under C or N-free conditions, the root growth was inhibited (Fig. 1A). However, relative to the shoot growth, the root growth displayed significant advantage under –N conditions whereas an opposite case was observed under –C conditions (Fig. 4B). We observed that –S conditions promote root branching (Fig. 1A) although an opposite observation was reported^56^. The analysis of shoot/root mass ratio suggested that –S relatively promotes root growth compared with the shoot growth (Fig. 4B). It has been revealed that plants employ miRNAs to alter the root system to adapt to the fluctuation of nutrient availability^57^. Nitrogen treatment led to a reduction in miR167 and the elevation of its target, *ARF8*, which mediates the balance between lateral root initiation and emergence^58^. miR160 controls root cap formation, lateral root number and primary root length by mediating the cleavage of its targets, *ARF10* and *ARF16*^40^. Both sequencing data and quantitative RT-PCR results indicated that miR160 was induced, whereas miR167 was suppressed, by –C, –N, and –S, implying that under nutrient deficiency conditions, or at least under –C, –N, and –S conditions, plants regulate root development-associated miRNAs to modulate their root systems. As expected, ectopic or disrupted expression of miR160 altered the root systems under both nutrient sufficiency and deficiency conditions (Fig. 4A). The analysis of relative root growth revealed that miR160 overexpression facilitated root growth under –C and –S conditions (Fig. 4B). miR395 plays key roles in modulating sulfate uptake and allocation by targeting genes encoding sulfate assimilation and transport proteins respectively^21^. Under –S conditions, elevation of miR395 resulted in advantageous root growth. In contrast, overexpression of miR399b inhibited primary root growth, but promoted later root initiation, under –N conditions. These results suggests that nutrient responsive miRNAs can regulate the root system of plants, which likely then affects uptake of nutrient elements.

To maintain normal growth and development, plants must acquire sufficient nutrients and keep the balance of nutrient elements, which are then incorporated into a variety of important compounds with structural and physiological roles. However, the different nutrient metabolic pathways are inter-dependent^59^. Deficiency of C induced miR169b/c, which was repressed by other nutrient deficiencies, including –N, –S, and –P. Similarly, –S upregulated miR395, which was downregulated by –C, –N, and –P. This nutrient-responsive pattern appears to be universal to miRNAs that are induced by a specific nutrient deficiency. For example, –Cu induced miRNAs (miR397, miR398, miR408, and miR857) that were suppressed by –C, –N, –S, and –P. miR399, miR827, and miR2111 were upregulated by –P, but downregulated by –C, –N, and –S. It implies that miRNAs that are induced specifically by a certain nutrient deficiency are often suppressed by other nutrient deficiencies, and that this likely prevents the imbalance of different nutrient elements in the plants because the downregulation of miRNAs can cause reduced uptake of the corresponding miRNA-associated nutrient elements. When miR395a-ox and miR399b-ox plants were subject to nutrient starvation treatments, they displayed significant morphological differences and altered adaptations to nutrient deficiencies, compared with wild-type plants (Fig. 4). Therefore, these nutrient deficiency-induced miRNAs may function as signals that mediate the crosstalk between nutrient metabolic pathways. Thus, it can be hypothesized that plants have developed a response to avoid imbalances in nutrient supply by modulating a suite of miRNAs that function in specific nutrient metabolic pathways.

## Methods

### Plant growth conditions

*Arabidopsis thaliana* (ecotype Columbia) seeds were used in this study. Sterilized seeds were suspended in 0.1% agarose and plated on MS medium. Plates were put in darkness for 2 d at 4 °C and then transferred to a tissue culture room at 22 °C under a 16-h-light/8-h-dark photoperiod.

For nutrient starvation experiments, plants were grown in long-day conditions on modified MS/agar media, containing 0.8% Agar A. For full nutrient, MS medium was used. For –C, the MS medium without the addition of sucrose was used. For –N, MS medium in which ammonium nitrate was removed and potassium nitrate was replaced with equivalent amounts of potassium chloride was used. For –S, the sulfate-containing salts of the MS medium were replaced with equivalent amounts of chloride salts. Root and shoot samples for RNA isolation were collected from 10-day-old seedlings grown vertically on MS medium.

### RNA preparation and small RNA sequencing

The Trizol reagent (Invitrogen) was used to extract total RNA. PEG8000/NaCl precipitation was used to isolate low molecular weight RNA from 200 μg total mRNAs. Small RNAs in the size range of 20 to 30 nucleotides were purified from 15% denaturing polyacrylamide gels and ligated first with the 5′ RNA adaptor and then with the 3′ RNA adaptor. At each step, the ligated products were purified by electrophoretic separation on polyacrylamide gels. After first-strand synthesis and 18 cycles of PCR amplification, the final bands were purified on PAGE gels and submitted for sequencing. The Beijing Genomics Institute (BGI) performed the sequencing.

### Computational analysis of sequencing data

The raw sequencing data were trimmed by removing adaptor sequences and mapped to the *Arabidopsis* genome (The *Arabidopsis* Information Resource release version 10; http://www.arabidopsis.org/). Reads perfectly matching those in the *Arabidopsis* genome were used for further analysis. *Arabidopsis* mature miRNAs and their precursors were retrieved from miRBase (http://www.mirbase.org).

### Construction of transgenic plants

The construction of miR160a-ox and miR395a-ox transgenic plants was reported previously^21^,^41^. For the construction of miR399b transgenic plants, a construct in which the CaMV35 promoter drove the expression of the DNA sequence containing the putative miR399b precursor was introduced into wild-type plants. For construction of STTM160 transgenic plants, a short tandem target mimic for miR160 was designed according to a previous report^60^.

### Gene expression analysis

Seedling samples were harvested separately from *Arabidopsis* and frozen in liquid nitrogen for storage at −80 °C. The Trizol reagent (Invitrogen) was used to isolate total RNA, which was digested by DNaseI (Fermentas). Stem-loop RT-PCR was used to detect the expression of miRNAs. To produce miRNA-fused stem-loop cDNA, 0.5 μg total RNA was used for reverse transcription with miRNA mature-sequence-specific stem-loop RT primers, according to the stem-loop RT-PCR protocol^61^. For mRNA cDNA, 1 μg of total RNA was reverse-transcribed using an oligo(dT)18 primer, according to the reverse transcription protocol (Takara). A 20-μl reaction mixture was used for the production of cDNA. After heat inactivation, a 1-μl aliquot was used as the template for real-time quantitative RT-PCR. An miRNA-specific primer and a universal primer were used to amplify miRNA-fused cDNA. Two specific primers were used to amplify each miRNA target gene. All primers used in this study are listed in Supplemental Table 6. *Arabidopsis ACT2* (*At3g18780*) was used as an internal control for real-time RT-PCR. A SYBR Premix Ex Taq^TM^ kit (TaKaRa) on a Roche LightCycler 480 real-time PCR machine performed all the quantitative RT-PCR analyses, according to the manufacturer’s instructions.

## Additional Information

**How to cite this article**: Liang, G. *et al*. Uncovering miRNAs involved in crosstalk between nutrient deficiencies in *Arabidopsis*. *Sci. Rep*. **5**, 11813; doi: 10.1038/srep11813 (2015).

## Supplementary Material

Supplementary Information

## Figures and Tables

**Table 1:** Summary statistics of small RNAs.

	FN				−C				−N				−S			
class	Unique sRNA	%	Total sRNA	%	Unique sRNA	%	Total sRNA	%	Unique sRNA	%	Total sRNA	%	Unique sRNA	%	Total sRNA	%
exon_antisense	48330	2.24%	153442	1.58%	43512	2.36%	113536	1.05%	47232	2.43%	151940	1.48%	51635	2.25%	142820	1.46%
exon_sense	92342	4.28%	199960	2.07%	89447	4.86%	168447	1.55%	99726	5.13%	194406	1.89%	99244	4.32%	198699	2.03%
intron_antisense	16696	0.77%	36220	0.37%	14950	0.81%	30837	0.28%	17120	0.88%	41205	0.40%	18643	0.81%	42446	0.43%
intron_sense	16028	0.74%	41067	0.42%	14763	0.80%	35726	0.33%	16174	0.83%	41399	0.40%	18370	0.80%	47541	0.48%
miRNA	3395	0.16%	3909038	40.38%	2850	0.15%	5344206	49.21%	2794	0.14%	3968586	38.58%	3092	0.13%	3378379	34.44%
rRNA	78278	3.63%	914877	9.45%	78036	4.24%	926000	8.53%	67848	3.49%	885685	8.61%	69497	3.03%	758028	7.73%
repeat	808137	37.43%	1671863	17.27%	641922	34.88%	1311398	12.07%	697382	35.90%	1627032	15.82%	856427	37.30%	1830010	18.65%
snRNA	2142	0.10%	4048	0.04%	1908	0.10%	3738	0.03%	3698	0.19%	10719	0.10%	2147	0.09%	4700	0.05%
snoRNA	1803	0.08%	3204	0.03%	1618	0.09%	3531	0.03%	2339	0.12%	7135	0.07%	2237	0.10%	6620	0.07%
tRNA	9187	0.43%	324707	3.35%	10844	0.59%	805859	7.42%	9780	0.50%	1040917	10.12%	8377	0.36%	841886	8.58%
unannotation	1082615	50.15%	2422924	25.03%	940557	51.11%	2117483	19.50%	978621	50.37%	2316339	22.52%	1166688	50.81%	2559578	26.09%
Total	2158953	100.00%	9681350	100.00%	1840407	100.00%	10860761	100.00%	1942714	100.00%	10285363	100.00%	2296357	100.00%	9810707	100.00%

**Table 2:** Summary of miRNAs commonly responsive to −C, −N, and −S and the potential functions in nutrient deficiency.

Family	Species		Expression			Target genes	Potential roles	References
			−C	−N	−S			
miR398	b,c	*	Down	Down	Down	Cu/Zn superoxide dismutase (CSD; CSD1, At1g08830; CSD2, At2g28190); copper chaperone for superoxide dismutase (CCS1, At1g12520); Cytochrome oxidase c(At3g15640)	Copper starvation response; Scavenge reactive oxygen species	23,24,33
miR397	a,b	*	Down	Down	Down	Laccase copper protein (LAC2,LAC4, LAC17, At2g29130, At2g38080, At5g60020)	Copper starvation response; Copper homeostasis	24
miR408		*	Down	Down	Down	Laccase copper protein (LAC3, LAC12, LAC13, At2g30210, At5g05390, At5g07130) Copper protein plantacyanin (At2g02850)	Copper starvation response; Copper homeostasis	24
miR857		*	Down	Down	Down	Laccase copper protein (LAC7, At3g09220)	Copper starvation response; Copper homeostasis	24
miR399	a,b,c,d,e,f	*	Down	Down	Down	Ubiquitin conjugase E2 (UBC24/PHO2; At2g33770)	Pi uptake and translocation	22
miR827		*	Down	Down	Down	Ubiquitin E3 ligase with RING and SPX domains (NLA/BAH1; At1g02860)	Nutrient recycle; Pi uptake and translocation	27
miR2111	a,b	*	Down	Down	Down	Kelch repeat-containing F-box protein (At3g27150)	Phosphate starvation response	28,29
miR775		*	Down	Down	Down	Galactosyltransferase family protein (At1g53290 )	Transferase activity, transferring hexosyl groups	36
miR172	a,b	*	Down	Down	Down	AP2 transcription factor (At5g60120, At4g36920, At2g28550, At2g28550, At5g67180)	Juvenile-to-adult transition	53
miR167	a,b,d	*	Down	Down	Down	Auxin response factor (ARF6, ARF8, At1g30330, At5g37020)	Root and pollen development; Stress responses	37,58
miR841		*	Down	Down	Down	Unknown	Unknown	
miR160	a,b,c	*	Up	Up	Up	Auxin response factor (ARF10, ARF16, ARF17, At2g283502, At4g300802, At1g778502)	Root growth and development; Stress response	40
miR169	b,c	@	Up	Down	Down	NFYA transcription factors (At1g17590, At1g54160, At1g72830, At3g05690, At3g20910, At5g06510, At5g12840)	Nitrogen homeostasis	25
miR837	3p	@	Down	Up	Up	Unknown	Unknown	
miR826		@	Down	Up	Down	Alkenyl hydroxalkyl producing 2(AOP2, At4g03060)	Nitrogen starvation response; Glucosinolate synthesis	26
miR395	a,b,c,d,e,f	@	Down	Down	Up	ATP sulfurylase (APS1, APS3, APS4; At3g22890, At4g14680, At5g43780) Sulfate transporter(SULTR2;1; At5g10180)	Sulfur homeostasis; Sulfate uptake and translocation	21

‘*’ indicates that the miRNAs are similarly regulated by three types of nutrient deficiencies (–C, −N, and –S) whereas ‘@’ indicates that the miRNAs are differentially regulated.
