# Author Correction: Uncovering miRNAs involved in crosstalk between nutrient deficiencies in *Arabidopsis*

**DOI:** 10.1038/s41598-020-62901-3

**Published:** 2020-04-20

**Authors:** Gang Liang, Qin Ai, Diqiu Yu

**Affiliations:** 1 Key Laboratory of Tropical Forest Ecology, Xishuangbanna Tropical Botanical Garden, Chinese Academy of Sciences, Kunming, Yunnan 650223 China; 2 University of Chinese Academy of Sciences, Beijing, 100049 China

Correction to: *Scientific Reports* 10.1038/srep11813, published online 02 July 2015

This Article contains errors. The authors analyzed four small RNA sequencing datasets, two of which were also been used in their previous paper [1] (cited as reference 41 in the present paper). Therefore, it is not appropriate to use the previous paper to support the observations in this paper. The sentence in the Results subsection ‘Expression correlation between miRNAs and their targets’,

“The expression patterns of miR826-AOP2 in –N and miR395-APS1/3/4 in –S agreed with the previous reports ^21,26,41^”

should read:

“The expression patterns of miR826-AOP2 in –N and miR395-APS1/3/4 in –S agreed with the previous reports ^21,26^”.

In addition, the information for the sequencing data is incomplete. The sequencing data is available in Sequence Read Archive of NCBI database under Accession numbers SRX7050411, SRX7050412, SRX7050413, and SRX7050414.
