# Supplementary material for: Uncovering miRNAs involved in crosstalk between nutrient deficiencies in Arabidopsis
Source: Sci Rep. 2015 Jul 2;5:11813. doi: 10.1038/srep11813 (PMC4488870; doi:10.1038/srep11813)
Supplement: Supplementary Information [file srep11813-s1.doc]

**Uncovering miRNAs involved in crosstalk between nutrient deficiencies in *Arabidopsis***

Gang Liang^1^, Qin Ai^1,2^, Diqiu Yu^1^*

^1^Key Laboratory of Tropical Forest Ecology, Xishuangbanna Tropical Botanical Garden, Chinese Academy of Sciences, Kunming, Yunnan 650223, China

^2^University of Chinese Academy of Sciences, Beijing 100049, China

| **Supplemental Table 1.** Differential expressed miRNAs. The miRNA abundance was normalized to transcripts per million (TPM) | | | | |
| --- | --- | --- | --- | --- |
| mature miRNA | FN | –C | –N | –S |
| miR156a | 140279 | 291586 | 169261 | 143207 |
| miR156b | 140290 | 291607 | 169273 | 143218 |
| miR156c | 140279 | 291586 | 169261 | 143207 |
| miR156d | 145317 | 302394 | 171766 | 148060 |
| miR156e | 140125 | 291355 | 169071 | 143092 |
| miR156f | 140126 | 291357 | 169071 | 143092 |
| miR156g | 610 | 1294 | 824 | 572 |
| miR156h | 48 | 45 | 303 | 52 |
| miR157a | 128737 | 94247 | 123606 | 102957 |
| miR157b | 128737 | 94247 | 123606 | 102957 |
| miR157c | 129292 | 94553 | 123473 | 103382 |
| miR157d | 4959 | 3522 | 2307 | 6901 |
| miR158a | 33123 | 27164 | 11526 | 22189 |
| miR158b | 338 | 182 | 285 | 215 |
| miR159a | 159 | 64 | 161 | 112 |
| miR159b | 58 | 20 | 53 | 50 |
| miR159c | 29 | 14 | 31 | 36 |
| miR160a | 46 | 72 | 274 | 86 |
| miR160b | 28 | 48 | 194 | 59 |
| miR160c | 46 | 72 | 275 | 87 |
| miR161.1 | 3389 | 2389 | 3192 | 3013 |
| miR161.2 | 3050 | 2275 | 1807 | 2160 |
| miR162a | 163 | 54 | 121 | 112 |
| miR162b | 163 | 54 | 122 | 112 |
| miR163 | 34 | 53 | 41 | 25 |
| miR164a | 1951 | 138 | 2072 | 1968 |
| miR164b | 1962 | 138 | 2079 | 1976 |
| miR164c | 219 | 259 | 177 | 369 |
| miR165a | 1339 | 794 | 3276 | 1284 |
| miR165b | 1309 | 781 | 3236 | 1259 |
| miR166a | 10670 | 15051 | 12717 | 9488 |
| miR166b | 10128 | 14296 | 11949 | 8983 |
| miR166c | 10141 | 14315 | 11962 | 8994 |
| miR166d | 10141 | 14315 | 11962 | 8994 |
| miR166e | 10130 | 14296 | 11957 | 8986 |
| miR166f | 10130 | 14296 | 11957 | 8986 |
| miR166g | 10142 | 14315 | 11970 | 8997 |
| miR167a | 42586 | 22518 | 26250 | 25629 |
| miR167b | 42630 | 22538 | 26270 | 25646 |
| miR167c | 86 | 62 | 349 | 118 |
| miR167d | 354 | 231 | 226 | 260 |
| miR168a | 5706 | 4906 | 6776 | 4328 |
| miR168b | 5701 | 4895 | 6770 | 4321 |
| miR169a | 270 | 307 | 93 | 145 |
| miR169b | 26 | 280 | 7 | 8 |
| miR169c | 21 | 293 | 5 | 5 |
| miR169d | 74 | 13 | 107 | 49 |
| miR169e | 74 | 13 | 107 | 49 |
| miR169f | 69 | 27 | 106 | 46 |
| miR169g | 69 | 23 | 105 | 46 |
| miR169h | 22 | 8 | 1 | 8 |
| miR169i | 67 | 24 | 6 | 24 |
| miR169j | 66 | 24 | 5 | 23 |
| miR169k | 22 | 8 | 1 | 8 |
| miR169l | 66 | 24 | 5 | 23 |
| miR169m | 24 | 8 | 1 | 9 |
| miR169n | 66 | 24 | 5 | 23 |
| miR170 | 3 | 6 | 4 | 4 |
| miR171a | 59 | 61 | 20 | 38 |
| miR171b | 8 | 5 | 13 | 5 |
| miR171c | 8 | 5 | 13 | 5 |
| miR172a | 1118 | 144 | 208 | 428 |
| miR172b | 1118 | 144 | 208 | 428 |
| miR172c | 7 | 2 | 13 | 11 |
| miR172d | 7 | 2 | 13 | 11 |
| miR172e | 28 | 3 | 51 | 30 |
| miR173 | 1668 | 947 | 2259 | 1177 |
| miR1886.2 | 51 | 51 | 40 | 39 |
| miR1888 | 19 | 13 | 2 | 8 |
| miR2111a | 20 | 0 | 2 | 7 |
| miR2111b | 20 | 0 | 2 | 7 |
| miR319a | 12 | 7 | 9 | 10 |
| miR319b | 7 | 3 | 9 | 5 |
| miR390a | 1171 | 627 | 1402 | 636 |
| miR390b | 1170 | 627 | 1400 | 635 |
| miR391 | 133 | 242 | 165 | 53 |
| miR393a | 5 | 4 | 4 | 8 |
| miR393b | 5 | 4 | 4 | 8 |
| miR394a | 25 | 17 | 26 | 31 |
| miR394b | 25 | 17 | 26 | 31 |
| miR395a | 5 | 0 | 1 | 3028 |
| miR395b | 8 | 0 | 0 | 356 |
| miR395c | 8 | 0 | 0 | 356 |
| miR395d | 5 | 0 | 1 | 3028 |
| miR395e | 4 | 0 | 1 | 2923 |
| miR395f | 8 | 0 | 0 | 356 |
| miR396a | 417 | 156 | 519 | 471 |
| miR396b | 312 | 123 | 382 | 349 |
| miR397a | 9 | 1 | 3 | 1 |
| miR397b | 79 | 4 | 2 | 13 |
| miR398b | 5 | 0 | 0 | 0 |
| miR398c | 5 | 0 | 0 | 0 |
| miR399a | 16 | 0 | 0 | 7 |
| miR399b | 123 | 2 | 0 | 72 |
| miR399c | 121 | 2 | 0 | 71 |
| miR399d | 11 | 0 | 0 | 5 |
| miR399e | 4 | 0 | 0 | 3 |
| miR399f | 36 | 0 | 0 | 17 |
| miR400 | 47 | 31 | 26 | 40 |
| miR402 | 83 | 75 | 94 | 88 |
| miR403 | 2026 | 1708 | 2025 | 2137 |
| miR408 | 73 | 5 | 19 | 27 |
| miR447a | 29 | 45 | 13 | 25 |
| miR447b | 29 | 45 | 13 | 25 |
| miR773 | 5 | 2 | 8 | 5 |
| miR775 | 460 | 199 | 147 | 227 |
| miR777 | 7 | 7 | 10 | 5 |
| miR822 | 3460 | 2493 | 2112 | 2716 |
| miR823 | 35 | 28 | 84 | 40 |
| miR824 | 56 | 44 | 113 | 78 |
| miR825 | 31 | 24 | 3 | 11 |
| miR826 | 0 | 0 | 9 | 0 |
| miR827 | 36 | 1 | 1 | 14 |
| miR829.1 | 66 | 23 | 358 | 81 |
| miR829.2 | 0 | 1 | 7 | 2 |
| miR833-3p | 1 | 1 | 1 | 2 |
| miR833-5p | 7 | 7 | 3 | 12 |
| miR837-3p | 58 | 9 | 124 | 94 |
| miR837-5p | 4 | 0 | 13 | 7 |
| miR841 | 18 | 8 | 1 | 7 |
| miR842 | 7 | 5 | 39 | 12 |
| miR843 | 17 | 111 | 9 | 13 |
| miR845a | 1 | 7 | 1 | 0 |
| miR846 | 87 | 107 | 360 | 148 |
| miR848 | 55 | 85 | 48 | 48 |
| miR850 | 23 | 27 | 0 | 8 |
| miR852 | 24 | 71 | 14 | 18 |
| miR857 | 21 | 1 | 1 | 3 |
| miR860 | 10 | 15 | 8 | 10 |
| miR863-3p | 282 | 372 | 9 | 141 |
| miR864-3p | 1 | 0 | 8 | 8 |
| miR866-5p | 10 | 18 | 27 | 14 |
| miR869.2 | 113 | 25 | 57 | 160 |

| **Supplemental Table 2.** Summary statistics of miR169 species. | | | | |
| --- | --- | --- | --- | --- |
| miRNA species | FN | –C | –N | –S |
| miR169a | 270 | 307 | 93 | 145 |
| miR169b,c | 26 | 293 | 7 | 8 |
| miR169d-g | 74 | 27 | 107 | 49 |
| miR169h-n | 67 | 24 | 6 | 24 |
| total | 437 | 651 | 213 | 226 |

**Supplemental Table 3.** Relative expression of miRNAs by analysis of real-time PCR.

| Family | Member | –C | |  | –N | |  | –S | |
| --- | --- | --- | --- | --- | --- | --- | --- | --- | --- |
|  |  | Mean | SE |  | Mean | SE |  | Mean | SE |
| miR398 | b,c | 0.02 | 0.01 |  | 0.03 | 0.006 |  | 0.01 | 0.006 |
| miR397 | a | 0.05 | 0.02 |  | 0.01 | 0.007 |  | 0.76 | 0.05 |
|  | b | 0.29 | 0.01 |  | 0.21 | 0.06 |  | 0.61 | 0.01 |
| miR408 |  | 0.49 | 0.06 |  | 0.62 | 0.04 |  | 0.84 | 0.07 |
| miR857 |  | 0.04 | 0.01 |  | 0.02 | 0.004 |  | 0.42 | 0.05 |
| miR399 | a | 0.01 | 0.005 |  | 0.07 | 0.03 |  | 0.62 | 0.06 |
|  | b,c | 0.04 | 0.002 |  | 0.02 | 0.01 |  | 0.53 | 0.04 |
|  | d | 0.05 | 0.01 |  | 0.12 | 0.07 |  | 0.54 | 0.01 |
|  | e | 0.81 | 0.02 |  | 0.56 | 0.01 |  | 0.91 | 0.03 |
|  | f | 0.73 | 0.03 |  | 0.73 | 0.03 |  | 1.25 | 0.08 |
| miR827 |  | 0.21 | 0.05 |  | 0.15 | 0.01 |  | 0.66 | 0.07 |
| miR2111 | a,b | 0.03 | 0.01 |  | 0.16 | 0.02 |  | 0.47 | 0.08 |
| miR775 |  | 0.15 | 0.04 |  | 0.24 | 0.08 |  | 0.58 | 0.04 |
| miR172 | a,b | 0.27 | 0.07 |  | 0.05 | 0.02 |  | 0.67 | 0.01 |
| miR167 | a,b | 0.71 | 0.01 |  | 0.62 | 0.04 |  | 0.81 | 0.01 |
|  | d | 0.83 | 0.09 |  | 0.51 | 0.08 |  | 0.69 | 0.04 |
| miR841 |  | 0.59 | 0.04 |  | 0.18 | 0.09 |  | 0.72 | 0.01 |
| miR160 | a,b,c | 4.92 | 0.04 |  | 9.82 | 0.08 |  | 2.8 | 0.09 |
| miR169 | b,c | 29.82 | 0.12 |  | 0.22 | 0.01 |  | 0.31 | 0.14 |
| miR837 | 3p | 0.63 | 0.09 |  | 2.12 | 0.02 |  | 1.87 | 0.03 |
| miR826 |  | 0.31 | 0.05 |  | 36.74 | 0.54 |  | 0.22 | 0.12 |
| miR395 | a,d,e | 0.21 | 0.07 |  | 0.13 | 0.01 |  | 181.12 | 2.64 |
|  | b,c,f | 0.14 | 0.06 |  | 0.23 | 0.03 |  | 63.24 | 0.87 |

**Supplemental Table 4.** Summary of miRNAs specifically responsive to –C, –N, and –S and the potential functions in nutrient deficiency.

| Nutrient | miRNA family | Expression | Target gene | AGI | Potential roles | Reference |
| --- | --- | --- | --- | --- | --- | --- |
| -C | miR163 | Up | PXMT1,FAMT | **At1g66700,At3g44860** | Secondary metabolite biosynthesis | Ng et al., 2011 |
|  | miR169b/c | Up | CAAT binding factor | **At1g17590,At1g54160,At1g72830,At3g05690,At3g20910,At5g06510,At5g12840** | Nitrogen homeostasis;Drought tolerance; | Zhao et al., 2011 |
|  | miR170 | Up | HAM1, HAM2, HAM3 | **At2g45160,At3g60630,At4g00150** | Maintenance of shoot and root indeterminacy | Llave et al., 2002 |
|  | miR391 | Up | TAS3 | **At3g17185** | Auxin response | Xia et al., 2013 |
|  | miR447 | Up | 2-phosphoglycerate kinase-related (2-PGK) | **At5g60760** | myo-inositol hexakisphosphate biosynthetic process | Allen et al., 2005 |
|  | miR843 | Up | Unknown | Unknown | Unknown |  |
|  | miR848 | Up | Unknown | Unknown | Unknown |  |
|  | miR159 | Down | MYB33,MYB65 | **At3g11440,At5g06100** | Male strility | Millar et al.,2005 |
|  | miR162 | Down | DCL1 | **At1g01040** | miRNA process | Xie et al., 2003 |
|  | miR164a/b | Down | NAC1,NAC2,CUC1,CUC2 | **At1g56010,At3g15510,At3g15170,At5g53950** | Leaf senescence;Lateral Root development; Embryonic, vegetative, and floral organ development | Guo et al., 2005;  Kim et al., 2009 |
|  | miR165 | Down | HD-ZIP III genes | **At1g30490,At2g34710,At5g60690** | Shoot apical meristem development | Williams et al., 2005 |
|  | miR169d-g | Down | CAAT binding factor | **At1g17590,At1g54160,At1g72830,At3g05690,At3g20910,At5g06510,At5g12840** | Nitrogen homeostasis;Drought tolerance; | Zhao et al., 2011  Li et al., 2008 |
|  | miR172c/d | Down | AP2 transcription factor | **At5g60120,At4g36920,At2g28550,At2g28550,At5g67180** | Juvenile-to-adult transition | Wu et al.,2009 |
|  | miR173 | Down | TAS1A,TAS1B,TAS1C,TAS2 | **At2g27400,At1g50055,At2g39675,At2g39681** | Unknown | Montgomery et al.,2008 |
|  | miR319 | Down | TCP familiy | **At1g30210,At1g53230,At2g31070,At3g15030,At4g18390** | Leaf Development | Jones-Rhoades et al,. 2004 |
|  | miR773 | Down | DNA methyltransferase, MET2 | **At4g14140** | Agrobacterium-mediated tumor formation | Fahlgren et al., 2007 |
|  | miR864-3p | Down | Unknown | Unknown | Unknown |  |
| -N | miR165 | Up | HD-ZIP III genes | **At1g30490,At2g34710,At5g60690** | Shoot apical meristem development | Williams et al., 2005 |
|  | miR167c | Up | Auxin response factor (ARF6,ARF8);IAR3 | **At1g30330,At5g37020,At1g51760** | Root and pollen development;Stress response | Wu et al., 2006; Gifford et al., 2008. Kinoshita et al., 2012 |
|  | miR171b/c | Up | HAM1, HAM2, HAM3 | **At2g45160,At3g60630,At4g00150** | Maintenance of shoot and root indeterminacy | Llave et al., 2002 |
|  | miR172c-e | Up | AP2 transcription factor | **At5g60120,At4g36920,At2g28550,At2g28550,At5g67180** | Juvenile-to-adult transition; | Wu et al.,2009 |
|  | miR823 | Up | Unknown | Unknown | Unknown |  |
|  | miR824 | Up | AGL16 | **At3g57230** | Stomatal Development; Flowering regulation | Fahlgren et al., 2007 |
|  | miR826 | Up | Alkenyl hydroxalkyl producing 2 | **At4g03060** | Nitrogen starvation response;Glucosinolate synthesis | He et al., 2014 |
|  | miR842 | Up | Mannose-binding lectin superfamily protein | **At5g49850**,At2g25980,At5g49870 | Unkown | Fahlgren et al., 2007 |
|  | miR829.1 | Up | Unknown | Unknown | Unknown |  |
|  | miR773 | Up | DNA methyltransferase,MET2 | **At4g14140** | Unknown | Fahlgren et al., 2007 |
|  | miR157d | Down | SPL transcription factors;HY5 | **At1g53160,At2g33810,At3g15270,At5g43270,At1g27360,At1g27370,At1g69170,At2g42200,At3g57920,At5g50570,At5g11260** | Juvenile-to-adult transition; Photomorphogenesis | Jones-Rhoades et al,. 2004; Wu et al.,2009; Tsai et al., 2014 |
|  | miR158a | Down | Glycosyltransferase | **At2g03210** | Glycosylation | German et al.,2008 |
|  | miR161.2 | Down | Pentatricopeptide repeat (PPR) | **At1g06580,At1g63150,At5g41170** | Unknown | Allen et al., 2004 |
|  | miR400 | Down | Unknown | Unknown | Unknown |  |
|  | miR447 | Down | 2-phosphoglycerate kinase-related (2-PGK) | **At5g60760** | myo-inositol hexakisphosphate biosynthetic process | Allen et al., 2005 |
|  | miR822 | Down | Unknown | Unknown | Unknown |  |
|  | miR833-5p | Down | Unknown | Unknown | Unknown |  |
|  | miR843 | Down | Unknown | Unknown | Unknown |  |
|  | miR852 | Down | Unknown | Unknown | Unknown |  |
| -S | miR164c | Up | NAC1,NAC2,CUC1,CUC2 | **At1g56010,At3g15510,At3g15170,At5g53950** | Leaf senescence;Lateral Root Development;Embryonic, vegetative, and floral organ development | Guo et al., 2005;  Kim et al., 2009 |
|  | miR395 | Up | ATP sulfurylase (APS1, APS3,APS4) | **At3g22890,At4g14680, At5g43780** | Sulfur homeostasis;Sulfate uptake and translocation | Jones-Rhoades et al,. 2004; Liang et al., 2010 |
|  |  |  | Sulfate transporter(SULTR2;1) | **At5g10180** |  |  |
|  | miR391 | Down | TAS3 | **At3g17185** | Auxin response | Fahlgren et al., 2007 |
|  | miR845a | Down | Unknown | Unknown | Unknown |  |

Boldface letters indicate the previously validated targets.

| **Supplemental Table 5.** Normalized abundance of miRNAs in Arabidopsis roots grown under –P. | | |
| --- | --- | --- |
| miRNA species | FN | –P |
| miR397b | 14 | 7 |
| miR398b | 20 | 2 |
| miR398c | 20 | 3 |
| miR408 | 522 | 173 |
| miR857 | 5 | 1 |
| miR395a | 7 | 1 |
| miR395b | 5 | 1 |
| miR395c | 5 | 1 |
| miR395d | 6 | 1 |
| miR395e | 7 | 1 |
| miR395f | 5 | 1 |
| miR169a | 1032 | 219 |
| miR169b | 461 | 152 |
| miR169c | 143 | 30 |
| miR169d | 236 | 47 |
| miR169e | 213 | 47 |
| miR169f | 233 | 50 |
| miR169g | 241 | 47 |
| miR169h | 121 | 12 |
| miR169i | 140 | 29 |
| miR169j | 131 | 24 |
| miR169k | 110 | 19 |
| miR169l | 127 | 26 |
| miR169m | 116 | 17 |
| miR169n | 123 | 26 |
| The data were retrieved from sequencing data by Hsieh et al., 2009 | | |

**Supplemental Table 6.** Primers used in this article.

qNFYA2-F:GCCTGTTCACAGCCAAAGCGGAT

qNFYA2-R:TCTCAGCCTAAGCCTCAGCAAAGT

qNFYA3-F:GTACCCTATCTTCATGAGTCTCGA

qNFYA3-R:AATGGTGCATGTCTCCACCTCCA

qNFYA5-F:GACTTGAGGCACATGAGAAGACCTT

qNFYA5-R:GGGTAATGCAATTTGTACTCTCAGA

qARF6-F:CAAAGTTTAGCAGCTACCACGA

qARF6-R:ACGTCGTTCTCTCGGTCAAC

qARF8-F:TTTGCTATCGAAGGGTTGTTG

qARF8-R:CATGGGTCATCACCAAGGA

qARF10-F: GGTTTCTCCGTTCCACGTTATT

qARF10-R: CCGTGGATGTCTTTAGCAATCA

qARF16-F:CGTTAAGCTCTGTTCTGGAC

qARF16-R:AGTAATGGTGAAGATCCGAAG

qARF17-F:GCACCTGATCCAAGTCCTTC

qARF17-R:GGTGAATAGCTGGGGAGGAT

qPHO2-F:CCCCTTTGAAGTTTATCCAACTGG

qPHO2-R:AGGTGAGCCAACTGAGGACTCC

qAPS1-F: AGGCTGGACAAGTCCACTCGG

qAPS1-R: GCCGTCGTCAAGACGTAGCGA

qAPS4-F: AGCGAAGGCTGGGCAAGTCC

qAPS3-F: GCGGCGGATTTGCCGAGAGT

qAPS3-R: CCCACGAAGAGGACTAGCCCAAC

qAPS4-R: AGCCGTCTTCGAGCCGGAAC

qLAC2-F: ACTGATGGTGAAACCTGGAAAGACG

qLAC2-R: CGCTCCTACGACCGTCAATGTATGA

qLAC3-F: TCGCTTTCCTCGCTTCTGCTGA

qLAC3-R: ACCACAAGCGTTGGACCAGGGT

qLAC4-F: ACGGACACCCAGGCCCAGTT

qLAC4-R: ACCGTGAAAATATGGCCGGCGA

qLAC12-F: AGAGACGCCGGTGAAGAGGCT

qLAC12-R: CTTCGAGCGTAGGCCCCGGA

qLAC13-F: AACGCCGGTGAAGAGGCTGT

qLAC13-R: AGGGAATCGCCGTTCCTCACCT

qLAC17-F: ACGATAAACGGGCTTCCTGGTCCA

qLAC17-R: ACCGTGTGATTTGCGATGCTGA

qNLA-F: ACAATTGTTCTCGTGAATGCCC

qNLA-R: GAGCATGCTCGTTAAACCATCC

qAOP2-F:AGAGGACAAGATACACAGCAGCA

qAOP2-R:AAGTCGCGGTAATCAAAAGGTC

qACT2-F:TGTGCCAATCTACGAGGGTTT

qACT2-R:TTTCCCGCTCTGCTGTTGT

miR160-RT: GTCGTATCCAGTGCAGGGTCCGAGGTATTCGCACTGGATACGACTGGCATA

miR160-F: GCATGCTGCCTGGCTCCCTGT

miR172a/b-RT: GTCGTATCCAGTGCAGGGTCCGAGGTATTCGCACTGGATACGACATGCAG

miR172a/b-F: GCGGCGAGAATCTTGATGATG

miR167a/b-RT: GTCGTATCCAGTGCAGGGTCCGAGGTATTCGCACTGGATACGACTAGATC

miR167d-RT:GTCGTATCCAGTGCAGGGTCCGAGGTATTCGCACTGGATACGACCCAGAT

miR167a/b/d-F: GCAGCCTGAAGCTGCCAGCAT

miR169bc-RT:GTCGTATCCAGTGCAGGGTCCGAGGTATTCGCACTGGATACGACCCGGCA

miR169bc-F: GCAGCCAGCCAAGGATGACT

miR395a/d/e-RT:GTCGTATCCAGTGCAGGGTCCGAGGTATTCGCACTGGATACGACGTGTTC

miR395b/c/f-RT:GTCGTATCCAGTGCAGGGTCCGAGGTATTCGCACTGGATACGACGTGTCC

miR395-F: GCACGTCTGAAGTGTTTGGGG

miR397a/b-RT:GTCGTATCCAGTGCAGGGTCCGAGGTATTCGCACTGGATACGACCATCAA

miR397a-F: GCGAGCTCATTGAGTGCAGCG

miR397b-F: GCGACGTCATTGAGTGCATCG

miR398b/c-RT: GTCGTATCCAGTGCAGGGTCCGAGGTATTCGCACTGGATACGACGTGTGT

miR398b/c-F: GCAGCGAGGGTTGATATGAGA

miR399a/b/c-RT: GTCGTATCCAGTGCAGGGTCCGAGGTATTCGCACTGGATACGACCAGGGC

miR399a/d/e/f-F: GCCGGCTGCCAAAGGAGATTT

miR399b/c-F: GCGACGTGCCAAAGGAGAGTT

miR399d-RT: GTCGTATCCAGTGCAGGGTCCGAGGTATTCGCACTGGATACGACCGGGGC

miR399e-RT: GTCGTATCCAGTGCAGGGTCCGAGGTATTCGCACTGGATACGACCGAGGC

miR399f-RT: GTCGTATCCAGTGCAGGGTCCGAGGTATTCGCACTGGATACGACCCGGGC

miR408-RT: GTCGTATCCAGTGCAGGGTCCGAGGTATTCGCACTGGATACGACCATGCT

miR408-F: GTCAGCACAGGGAACAAGCAG

miR775-RT: GTCGTATCCAGTGCAGGGTCCGAGGTATTCGCACTGGATACGACTGGCTC

miR775-F: GGCAGCTTCGATGTCTAGCA

miR826-RT:GTCGTATCCAGTGCAGGGTCCGAGGTATTCGCACTGGATACGACCACGTA

miR826-F:GCAGCCTAGTCCGGTTTTGGA

miR827-RT: GTCGTATCCAGTGCAGGGTCCGAGGTATTCGCACTGGATACGACAGTTTG

miR827-F: GGCGCGUUAGAUGACCAUCAA

miR837-RT:GTCGTATCCAGTGCAGGGTCCGAGGTATTCGCACTGGATACGACCCATCA

miR837-F: GGCGCGAAACGAACAAAAAAC

miR841-RT: GTCGTATCCAGTGCAGGGTCCGAGGTATTCGCACTGGATACGACTTCAGT

miR841-F: GACGCTACGAGCCACTTGAA

miR857-RT: GTCGTATCCAGTGCAGGGTCCGAGGTATTCGCACTGGATACGACATACAC

miR857-F: GCGGCGTTTTGTATGTTGAAG

miR2111-RT: GTCGTATCCAGTGCAGGGTCCGAGGTATTCGCACTGGATACGACTAAACC

miR2111-F: GGCAGCTAATCTGCATCCTGA

Universal: GTGCAGGGTCCGAGGT

Pre-miR399b_F: GAGCTCcagacacaagccttcatatgg

Pre-miR399b_R: GGATCCgaagaggaagagtgtacgtac

Sttm160-F: aaGAGCTCtggcatacaggctagagccaggcaGTTGTTGTTGTTATGGTCTAATTTAAATATGGTC

Sttm160-R: aaGGATCCtgcctggctctagcctgtatgccaATTCTTCTTCTTTAGACCATATTTAAATTAGACC

**References:**

**Allen E, Xie Z, Gustafson AM, Sung GH, Spatafora JW, Carrington JC.** 2004. Evolution of microRNA genes by inverted duplication of target gene sequences in Arabidopsis thaliana. *Nature Genetics* **36,**1282-1290.

**Allen E, Xie Z, Gustafson AM, Carrington JC.** 2005. microRNA-directed phasing during trans-acting siRNA biogenesis in plants. *Cell* **121,** 207-221.

Fahlgren N, Howell MD, Kasschau KD, Chapman EJ, Sullivan CM, Cumbie JS, Givan SA, Law TF, Grant SR, Dangl JL, Carrington JC. 2007. High-throughput sequencing of Arabidopsis microRNAs: evidence for frequent birth and death of MIRNA genes. *PLoS One* **14,**2(2):e219.

**German MA, Pillay M, Jeong DH, Hetawal A, Luo S, et al.** 2008. Global identification of microRNA-target RNA pairs by parallel analysis of RNA ends. *Nature Biotechnology* **8,** 941–946.

**Gifford ML, Dean A, Gutieerez RA, Coruzzi GM, Birnbaum KD.** 2008. Cell-specific nitrogen responses mediate developmental plasticity. *Proceedings of the National Academy of Sciences of the United States of America* **105,** 803–808.

**Guo HS, Xie Q, Fei JF, Chua NH.** 2005. MicroRNA directs mRNA cleavage of the transcription factor NAC1 to downregulate auxin signals for arabidopsis lateral root development. *Plant Cell* **17,** 1376-1386.

**He H, Liang G, Li Y, Wang F, Yu D.** 2014. Two young MicroRNAs originating from target duplication mediate nitrogen starvation adaptation via regulation of glucosinolate synthesis in Arabidopsis thaliana. *Plant Physiology* **164,** 853-865.

**Jones-Rhoades MW, Bartel DP** .2004. Computational identification of plant microRNAs and their targets, including a stress-induced miRNA. *Molecular Cell* **14,** 787–799.

**Kim JH, Woo HR, Kim J, Lim PO, Lee IC, Choi SH, Hwang D, Nam HG.** 2009. Trifurcate feed-forward regulation of age-dependent cell death involving miR164 in Arabidopsis. *Science* **323,** 1053-1057.

**Kinoshita N, Wang H, Kasahara H, Liu J, Macpherson C, Machida Y, Kamiya Y, Hannah MA, Chua NH.** 2012. IAA-Ala Resistant3, an evolutionarily conserved target of miR167, mediates Arabidopsis root architecture changes during high osmotic stress. Plant Cell 24, 3590-3602.

**Li WX, Oono Y, Zhu J, He XJ, Wu JM, Iida K, Lu XY, Cui X, Jin H, Zhu JK.** 2008.The Arabidopsis NFYA5 transcription factor is regulated transcriptionally and posttranscriptionally to promote drought resistance. *Plant Cell* **20,** 2238-2251.

**Liang G, Yang FX, Yu DQ.** 2010. MicroRNA395 mediates regulation of sulfate accumulation and allocation in Arabidopsis thaliana. *The Plant Journal* **62,** 1046–1057.

**Llave C, Xie Z, Kasschau KD, Carrington JC.** 2002. Cleavage of Scarecrow-like mRNA targets directed by a class of Arabidopsis miRNA. *Science* **297**, 2053-2056.

**Millar AA, Gubler F.** 2005. The Arabidopsis GAMYB-like genes, MYB33 and MYB65, are microRNA-regulated genes that redundantly facilitate anther development. *Plant Cell* **17,** 705-721.

**Montgomery TA, Yoo SJ, Fahlgren N, Gilbert SD, Howell MD, Sullivan CM, Alexander A, Nguyen G, Allen E, Ahn JH, Carrington JC.** 2008. AGO1-miR173 complex initiates phased siRNA formation in plants. *Proceedings of the National Academy of Science the United States of America*. **105,** 20055-20062.

**Ng DW, Zhang C, Miller M, Palmer G, Whiteley M, Tholl D, Chen ZJ.** 2011. cis- and trans-Regulation of miR163 and target genes confers natural variation of secondary metabolites in two Arabidopsis species and their allopolyploids. *Plant Cell* **23,**1729-1740.

**Tsai H, Li Y, Hsieh W, Lin M, Ahn JH, Wu S.** 2014. HUA ENHANCER1 is involved in posttranscriptional regulation of positive and negative regulators in Arabidopsis photomorphogenesis. *Plant Cell* doi: 10.1105/tpc.114.126722.

**Xia R, Meyers BC, Liu Z, Beers EP, Ye S, Liu Z.** 2013. MicroRNA superfamilies descended from miR390 and their roles in secondary small interfering RNA Biogenesis in Eudicots. *Plant Cell* **25,**1555-1572.

**Xie Z, Kasschau KD, Carrington, JC.** 2003. Negative feed-back regulation of Dicer-Like1 in Arabidopsis by microRNA-guided mRNA degradation. *Current Biology* **13,** 784–789.

**Williams L, Grigg SP, Xie M, Christensen S, Fletcher JC.** 2005. Regulation of Arabidopsis shoot apical meristem and lateral organ formation by microRNA miR166g and its AtHD-ZIP target genes. *Development* **132,** 3657-3668.

**Wu G, Park MY, Conway SR, Wang JW, Weigel D, Poethig RS.** 2009. The sequential action of miR156 and miR172 regulates developmental timing in Arabidopsis. *Cell***138,** 750-759.

**Wu MF, Tian Q, Reed JW.** 2006.Arabidopsis microRNA167 controls patterns of ARF6 and ARF8 expression, and regulates both female and male reproduction. *Development* **133,** 4211-4218.

**Zhao M, Ding H, Zhu JK, Zhang F, Li WX**. 2011. Involvement of miR169 in the nitrogen-starvation responses in Arabidopsis. *New Phytologist* **190,** 906–915.
